# Electrophysiological signature of stroke recovery: investigating EEG biomarkers for prognostic insights

**DOI:** 10.3389/fstro.2026.1894659

**Published:** 2026-09-28

**Authors:** Majid Khalili-Ardali, Vivek Sharma, Tarandeep Singh Mandahar, Francisco Páscoa dos Santos, Paul Tiesinga, Nick Ramsey

**Affiliations:** 1 Donders Institute for Brain, Cognition, and Behaviour, Radboud University, Nijmegen, Netherlands; 2 Cognitive and Systems Neuroscience Group, Swammerdam Institute for Life Sciences, Faculty of Science, University of Amsterdam, Amsterdam, Netherlands

**Keywords:** EEG, lesion, metastability, microstates, stroke, synchrony

## Abstract

**Introduction:**

Stroke is a leading cause of long-term disability, often resulting in persistent motor impairments that reflect disruptions in large-scale brain networks. An integrated framework capturing spatiotemporal EEG dynamics would help clarify how these networks change during recovery.

**Methods:**

We analysed resting-state EEG from stroke patients at 1 week (Session 1) and three months (Session 2) post-stroke to investigate electrophysiological biomarkers of motor recovery, indexed by change in the Fugl-Meyer scale (Δ*FM*). We quantified relative alpha-band power, microstate metrics (mean duration, complexity, and transition probabilities), and measures of metastability and synchrony derived from the Kuramoto Order Parameter.

**Results:s:**

Change in relative alpha power emerged as the strongest single correlate of motor improvement, accounting for the largest proportion of variance among the EEG measures examined. Metastability and synchrony did not individually reach statistical significance but showed moderate positive correlations with Δ*FM*, particularly in the alpha and theta bands, and added 26% explained variance in Δ*FM* when combined with alpha power. Microstate parameters did not explain additional variance once alpha power and network-level dynamics were accounted for. A hierarchical model combining alpha power, metastability/synchrony, and microstates explained over 78% of the variance in Δ*FM*.

**Discussion:**

These findings indicate that stroke recovery involves restoring balanced alpha oscillations and flexible large-scale brain coordination. Larger samples and more frequent longitudinal assessments are required to confirm the prognostic utility of integrated EEG biomarkers for guiding personalised stroke rehabilitation strategies.

## 1 Introduction

It is widely recognized that at rest the brain remains highly active, displaying intricate and ever-changing spatiotemporal patterns of activity (Raichle et al., 2001; Fox, 2010; Biswas and Banerjee, 2026). This spontaneous neural activity is fundamental to brain function, as it predicts behavior and reflects intrinsic network dynamics (Cabral et al., 2014). However, brain injuries such as stroke disrupt these network dynamics, leading to impairments in sensory, motor, and cognitive functions (Salvalaggio et al., 2020). Motor impairments, in particular, are the most common consequence of stroke, affecting up to 85% of patients in the acute phase and significantly limiting their autonomy in daily activities (Bajaj et al., 2015; Baldassarre et al., 2016; Ramsey et al., 2017). Understanding how stroke alters large-scale brain dynamics could be of significant value for identifying neural markers of recovery and optimizing rehabilitation strategies.

Neurophysiological approaches such as electroencephalography (EEG) provide insights into stroke-related brain dysfunction and altered temporal dynamics of neural activity (Khanna et al., 2015). Quantitative EEG measures have shown prognostic potential in stroke. For instance, global slowing of EEG rhythms—often indexed by a high delta-to-alpha power ratio—correlates with acute stroke severity and worse outcomes (Sheorajpanday et al., 2011). Likewise, interhemispheric asymmetry in alpha power (quantified by the Brain Symmetry Index) has been linked to motor impairment, with more asymmetric alpha indicating poorer upper extremity function (Wang et al., 2022). Beyond traditional spectral and asymmetry measures, more recent analytical approaches have expanded our ability to characterize dynamic brain states. One such well-established method is EEG microstate analysis (Lehmann, 1987), which identifies quasi-stable topographical patterns in scalp electrical activity, which are thought to represent transient global network states (Koenig et al., 2002; Michel and Koenig, 2018). These microstates, which typically last between 40 and 120 ms, are thought to reflect fundamental neural processes underlying cognition and behavior (Britz et al., 2010; Pascual-Marqui et al., 1995). While microstate analysis has been extensively applied to psychiatric and neurodegenerative disorders such as schizophrenia and Alzheimer's disease (Tait et al., 2020; Musaeus et al., 2019), its potential in understanding stroke recovery has only recently begun to be explored. Early work has pointed out that acute phase microstate patterns might have prognostic value in stroke (Zappasodi et al., 2017). Additionally, it has been proposed that subacute stroke patients exhibit distinct microstate characteristics in comparison with healthy controls, and the microstate metrics (such as duration, transition probabilities, and complexity) correlate with motor function and recovery (Wang et al., 2022; Hao et al., 2022).

Beyond spatiotemporal correlates, metastability—the balance between stable and flexible brain states — has emerged as a critical marker of functional integration and segregation in brain networks (Hancock et al., 2025; Deco et al., 2015). Metastability describes the brain's ability to transition between transiently stable states while maintaining dynamic flexibility, a feature essential for adaptive behavior and recovery following brain injury (Kelso, 2012; Cabral et al., 2017). Recent advances suggest that alterations in metastability may provide insights into network reorganization after stroke and could serve as an indicator of functional recovery (Hancock et al., 2025; Shanahan, 2010). Another key measure of large-scale brain dynamics is synchrony, which refers to the temporal coordination of neural oscillations across different brain regions (Deco et al., 2015). Synchrony plays a crucial role in motor control and sensorimotor integration, and disruptions in synchrony following stroke have been linked to motor deficits (Trujillo et al., 2017). In particular, a reduction in interhemispheric alpha coherence has been associated with poorer motor function in daily activities (Kawano et al., 2017). In contrast, it has been suggested that abnormally high intrahemispheric connectivity may reflect minimal coupling that limits motor recovery (Hoshino et al., 2020). This growing evidence on the importance of large-scale dynamical features of EEG recordings points to how EEG synchrony measures could indicate how network integration (or its imbalance) contributes to motor impairment and may provide a more comprehensive understanding of stroke-related functional changes and inform rehabilitation strategies.

These findings underscore that EEG can sensitively reflect functional deficits post-stroke. Although previous studies have examined microstate dynamics, metastability, synchrony, and spectral features separately, a unified framework that integrates the large-scale network dynamics with the spatiotemporal pattern of EEG activity and traditional spectral features is still lacking. In this study, we analyse resting-state EEG in stroke patients to investigate changes in microstate dynamics, metastability, synchrony, and classical spectral measures as potential markers of motor recovery. Specifically, we examine how microstate characteristics—such as duration, occurrence, and transition probabilities—are modulated after stroke and whether these changes correlate with functional recovery. Given that microstates reflect transient states of large-scale neural networks, their disruption may provide critical insights into altered brain dynamics post-stroke. Additionally, we assess metastability and synchrony, which capture the temporal stability and coordination of neural oscillations, respectively. While spectral measures are well-established in clinical EEG research, spatiotemporal features (as measured by microstate analysis) and large-scale network dynamics (as measured by metastability and synchrony analysis), despite extensive modeling studies, remain less investigated in clinical applications for stroke populations. Studying these dynamics in clinical settings could yield novel insights into network reorganization during recovery. Our study aims to characterize each metric independently (microstate parameters, metastability, synchrony, and spectral features) and to develop a unified multivariate model which integrates all these features to better understand the neurophysiological mechanisms underlying stroke rehabilitation. These findings could contribute to the development of more effective therapeutic interventions, ultimately guiding personalized rehabilitation strategies based on neurophysiological biomarkers.

## 2 Methodology

### 2.1 Data acquisition

A total of 20 stroke patients with a first-ever unilateral ischemic stroke in the territory of the middle and/or anterior cerebral artery were enrolled in this cohort. Three patients were excluded due to missing follow-up scores, leaving 17 patients for final analysis; all demographic and clinical figures reported below refer to this final sample of 17 unless stated otherwise. Electrophysiological and behavioral data were collected at two time points: one week post-stroke onset (Session 1) and three months post-stroke onset (Session 2). All strokes were ischaemic, with no haemorrhagic cases included. Age ranged from 37 to 79 years (mean 58.9, 7 females). Stroke severity at admission, indexed by the National Institute of Health Stroke Scale (NIHSS), ranged from 3 to 27 (mean 12.1), indicating that the cohort spanned mild to severe strokes rather than a narrow severity band. Lesion side was approximately balanced across the cohort (10 left-sided, 7 right-sided), and stroke etiology was heterogeneous, including large-artery disease (e.g. internal carotid artery stenosis, dissection, or occlusion), cardioembolic sources (e.g. atrial fibrillation, patent foramen ovale), and cryptogenic strokes. The original 20-patient cohort is described in Dubovik et al. (2012). Absolute Recovery (AR), as a measure of improvement and functional recovery, was extracted from the normalized Fugl-Meyer (FM) scale (Fugl-Meyer et al., 1975). For each patient, the raw FM score was normalized to the maximum score achievable and computed as the difference (Session 2-Session 1) between the normalized FM. Because each participant was assessed at only two time points, the primary longitudinal variables were defined as session-difference scores (Session 2-Session 1) for both EEG measures and contralesional Fugl-Meyer motor scores. This approach directly tested whether inter-individual differences in EEG change over the recovery interval were associated with inter-individual differences in motor change over the same interval. Although repeated-measures correlation can estimate within-individual associations across repeated paired measurements (Bakdash and Marusich, 2017), and mixed-effects models are useful for longitudinal datasets with multiple observations per participant, the present two-time-point design provides only one within-subject change trajectory per participant and therefore limits the additional information that can be gained from more flexible longitudinal modeling. Since subjects were largely functionally intact on the ipsilesional side, we only used the Fugl-Meyer motor scores for the contralesional side relative to the lesion.

EEG was recorded using a 128-channel Biosemi ActiveTwo EEG-system (Biosemi B.V., Amsterdam, Netherlands) at a sampling rate of 512 Hz while participants rested awake with their eyes closed. The dataset used in this study was provided by Adrian Guggisberg and made available through the AISN consortium framework, and was previously published in Dubovik et al. (2012, 2013). The present work did not include new data collection or interaction with human participants. For full details of the original data acquisition, including ethical approval and informed consent, consult (Dubovik et al., 2012).

### 2.2 EEG data preprocessing

The EEG preprocessing was carried out in Matlab code (R2023a; The MathWorks, Natick, MA, United States) using the Fieldtrip toolbox (Oostenveld et al., 2011). We applied a zero-phase, two-pass, 4th-order Butterworth IIR filter with a passband from 0.5 to 45 Hz, which was followed by detrending the signal and a notch filter to remove the line noise at 50 Hz. Noisy segments of the data were rejected by visual inspection and using quality metrics (e.g., kurtosis, db amplitude, variance, 1/variance, and z-score), followed by common average referencing (Oostenveld et al., 2011). Rejected segments never exceeded 10 s in length, with total rejection per subject remaining below 1 min, leaving at least 3 min of clean data. All 128 channels were preprocessed, and bad channels were interpolated using nearest neighbor referencing. Approximately 10% of electrodes were labeled as bad channels. We applied *fitting oscillations* and *one over f* (FOOOF) on the power spectral densities (PSD) extracted from the preprocessed EEG data to remove the 1/f power and only analyse the oscillatory part of the signal (Gerster et al., 2022). All the subsequent analysis steps were performed on this periodic component only. For the frequency band ranges, we used delta (0.5–4 Hz), theta (4–8 Hz), alpha (8–13 Hz), and beta (13–30 Hz) (Cabral et al., 2022). The relative band powers were calculated as the area under the curve in the specific band range divided by the total power and correlated with the Δ*FM* using Pearson correlation coefficients (Oostenveld et al., 2011).

### 2.3 Microstates

We used the +microstate toolbox for calculating the EEG microstates (Tait and Zhang, 2022), which uses a modified k-means clustering method, based on topographical dissimilarity instead of the Euclidean distance. First, the standard deviation of the voltages across all channels at each time point, known as the Global Field Power (GFP), was extracted from the preprocessed EEG data as follows:

$$\begin{array}{l} GFP=\sqrt{\frac{\overset{N}{\underset{i=1}{\sum }}{\left(u_{i}-ū\right)}^{2}}{N}} \end{array}$$ (1)

Where *u*_*i*_ is the voltage at electrode *i*, ${\bar{u}}_{i}$ is the average voltage of all electrodes, and N is the number of electrodes. Once the GFP was obtained for the entire EEG recording, topographic maps at the local maxima of the GFP in time were clustered via a modified k-means algorithm. We determined the optimal number of clusters to be six using the silhouette score (Rousseeuw, 1987). Real-time EEG microstates were then derived *post hoc* by fitting these cluster maps back to each time point in the data, and the statistical significance of the classification accuracy was tested with 100 permutations, for details see Michel and Koenig (2018). Because lesion side, location, and recovery severity varied among patients, and because we hypothesize that microstate parameters correlate with network dynamics and recovery, microstates were computed individually for each subject and session. This approach assumes that microstates can change significantly post-stroke and helps account for inter-subject variability. Figure 1 shows a sample of one-second EEG data segmentation into 6 microstates.

**Figure 1:**
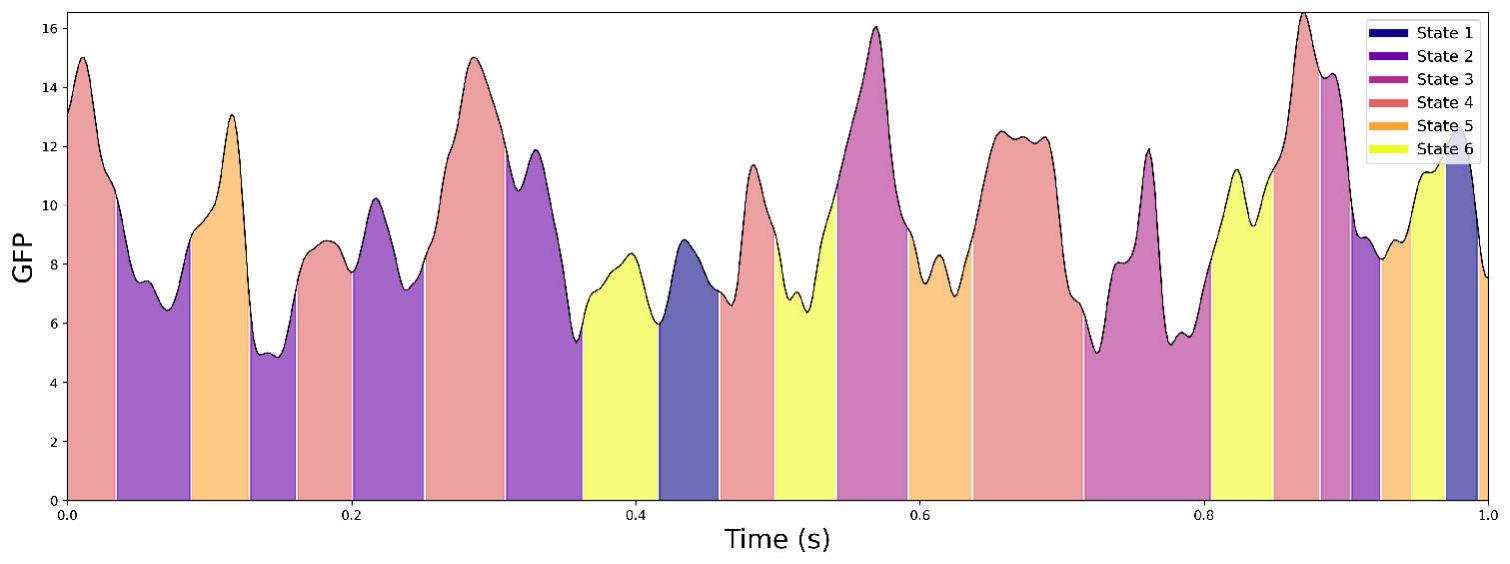
EEG Microstates Segmentation. Global Field Power (GFP) time series overlaid with corresponding microstate segmentation. Each color-coded segment indicates a distinct microstate cluster derived from spatial topography. Note that GFP reflects the global variance across channels at each time point and does not necessarily distinguish between microstates, which are defined by spatial configuration rather than amplitude differences. Repeated GFP ranges across states reflect distinct spatial patterns occurring at similar global variance levels.

Once the microstate time series was calculated for each subject and session, six measures were derived and correlated separately with the Δ*FM* using Pearson correlation coefficients: (1) the average microstate duration, (2) the Hurst exponent—an index of long-range correlations in time-series data (Van De Ville et al., 2010), (3) the Lempel—Ziv-inspired complexity—capturing the algorithmic or sequence complexity (Tait et al., 2020), (4) the GFP peak frequency, (5) the global explained variance (GEV)—which indicates how much of the original EEG signal is explained by the identified microstates (Hu et al., 2022), and (6) the state transition probability matrix. For the transition probability matrix, the average duration of all microstates (computed separately per subject and session) served as a duration threshold; a transition from the current state to the next (including the same state) was counted whenever the time series remained in one state for at least this threshold. Only transitions with probabilities exceeding those of a baseline model are displayed in Figure 2 and Supplementary Figure S1. This baseline model was generated by performing 1,000 permutations of the transition matrices for each subject and session, creating a null distribution to assess statistical significance. Once we obtain the null distribution after running these permutations, we proceed to compute the significant differences in the observed transition probability matrix, as compared to the null distribution. For each transition probability *tp*(*i, j*) in the observed 6 × 6 transition matrix (representing transitions between 6 microstates), we compute the null mean (expected transition probability under the null hypothesis) and standard deviation as:

**Figure 2:**
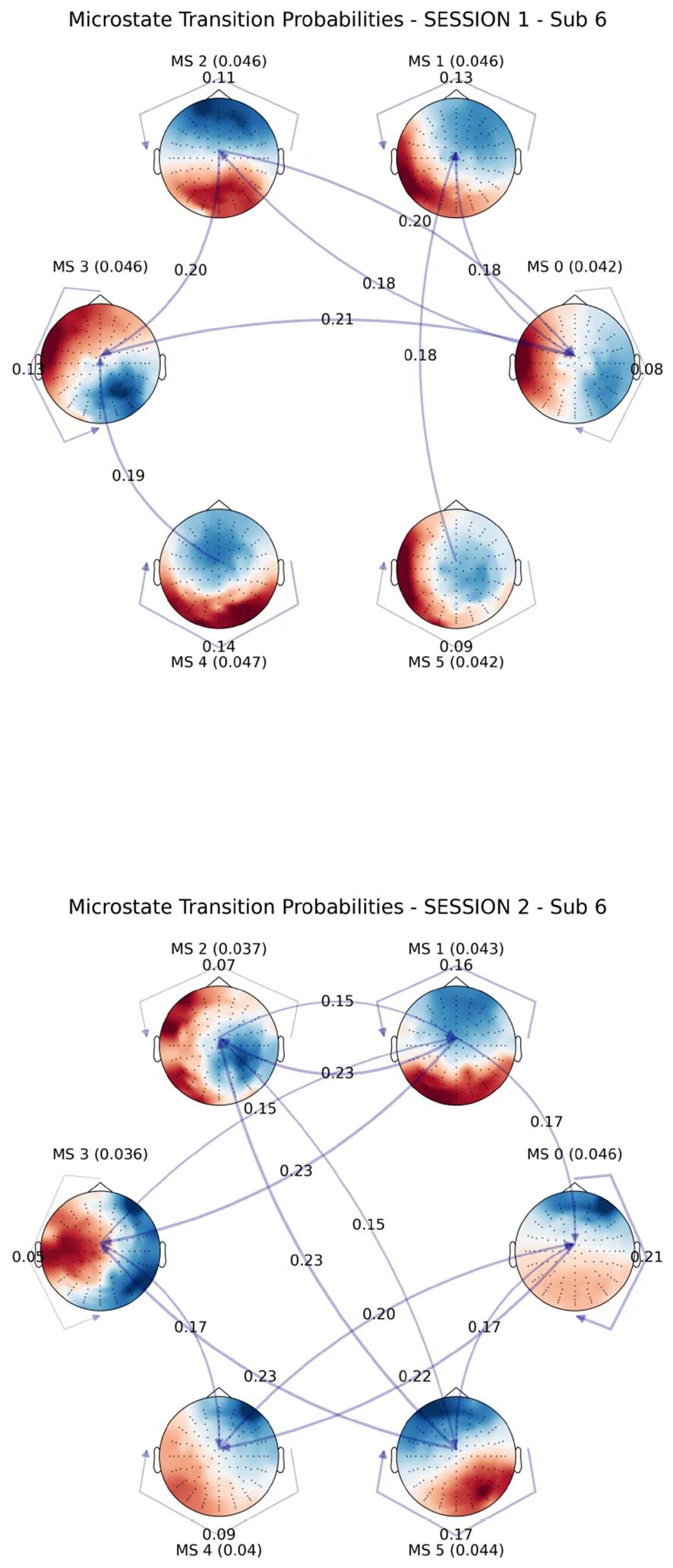
Transition Probabilities for subject 6. Directed graphs showing microstate transition probabilities for Session 1 and Session 2. Microstates were independently extracted for each session, reflecting the expected variability due to lesion effects and recovery over time. As such, microstate labels (MS1–MS6) may not correspond directly between sessions. The number of states was kept constant at six per session, based on silhouette distance optimization, to enable comparable transition structures. Only transitions that were significantly above chance level, determined via bootstrap resampling, are shown.


$$\begin{array}{l} \begin{array}{c} \text{null\_mean}\left[i,j\right]=\frac{1}{N}\overset{N}{\underset{k=1}{\sum }}\text{perm\_tp}\left[k,i,j\right],\\ \text{null\_std}\left[i,j\right]=\sqrt{\frac{1}{N-1}\overset{N}{\underset{k=1}{\sum }}{\left(\text{perm\_tp}\left[k,i,j\right]-\text{null\_mean}\left[i,j\right]\right)}^{2}}. \end{array} \end{array}$$ (2)

where *N* = 1, 000 is the number of permutations and perm_tp[*k, i, j*] is the transition probability for states *i* to *j* in the *k*-th permutation.

We then calculate the z-score for each transition, assuming the null distribution is approximately normal:

$$\begin{array}{l} z\left(i,j\right)=\frac{\text{obs\_tp}\left[i,j\right]-\text{null\_mean}\left[i,j\right]}{\text{null\_std}\left[i,j\right]} \end{array}$$ (3)

where obs_tp[*i, j*] is the observed transition probability.

To account for multiple comparisons across the 36 transitions in the 6 × 6 matrix, we apply a Bonferroni correction to control the family-wise error rate. With a nominal significance level of α = 0.05, the adjusted threshold is α_bonf_ = α/36 ≈ 0.00139. Transitions with *p*(*i, j*) < α_bonf_ are deemed statistically significant.

### 2.4 Metastability and synchrony

To analyse oscillatory dynamics, EEG signals were bandpass filtered into four standard frequency bands: delta (0.5–4 Hz), theta (4–8 Hz), alpha (8–13 Hz), and beta (13–30 Hz). The filtering process was applied separately for each trial using a second-order Butterworth bandpass filter implemented with zero-phase forward and reverse filtering to prevent phase distortions (Gustafsson, 1996). Prior to bandpass filtering, the broadband signals were orthogonalized across all 128 channels using sequential Gram-Schmidt orthogonalization, in which each channel was orthogonalized with respect to all preceding channels in the recording montage, to reduce spatial leakage between electrodes (Nolte et al., 2004; Stam et al., 2007). The orthogonalized signals were then bandpass filtered into four standard frequency bands and subjected to a Hilbert transform to extract instantaneous phase information, and the amplitude of the analytic signal was normalized to obtain pure phase values.

Metastability and synchrony were assessed using the Kuramoto Order Parameter (KOP), a well-established measure of global phase synchrony (Hancock et al., 2025). The KOP at each time point quantifies the degree of phase alignment across electrodes and is computed by averaging the unit-norm complex exponentials of the phase angles (Hancock et al., 2025; Cabral et al., 2022).

The Kuramoto Order Parameter at time *t* is given by:

$$\begin{array}{l} R\left(t\right)=|\frac{1}{N}\overset{N}{\underset{j=1}{\sum }}e^{i\theta_{j}\left(t\right)}| \end{array}$$ (4)

where θ_*j*_(*t*) represents the phase of electrode *j* at time *t*, and *N* is the total number of electrodes.

To characterize network dynamics, the mean KOP value over time was used as a measure of global synchronization, while its standard deviation captured the degree of temporal variability in synchrony, reflecting metastability (Shanahan, 2010).

For each subject and frequency band, the KOP was calculated by computing the phase of each orthogonalized and filtered signal, averaging the unit norm complex exponentials of phase angles across EEG channels, extracting the absolute value of the resulting vector to obtain a time series of phase synchrony values and computing the mean (synchrony) and standard deviation (metastability) of the KOP time series.

To examine the relationship between metastability, synchrony, and motor recovery, the differences in these measures were computed across the two recording sessions. These differences were then correlated with changes in Fugl-Meyer motor scores, a clinical measure of motor recovery. Pearson correlation was used to quantify the strength of the relationship between these variables, computed using the formula:

$$\begin{array}{l} R_{ij}=\frac{C_{ij}}{\sqrt{C_{ii}C_{jj}}} \end{array}$$ (5)

where *C*_*ij*_ represents the covariance between variables *i* and *j*, and *C*_*ii*_ and *C*_*jj*_ are the variances of each variable. Pearson correlation coefficients were extracted from the correlation matrix to evaluate the association between changes in EEG-derived measures and motor recovery outcomes.

### 2.5 Unified multivariate framework

We developed a three-block hierarchical regression model to integrate EEG measures from multiple domains into a single explanatory framework for motor recovery overall model *p* = 0.0010 (Table 1). To reduce dimensionality in this small-sample dataset, we constructed one summary predictor for each EEG domain rather than entering all individual metrics into the same regression model. In the spectral domain, we used relative alpha power change. In the network-level domain, synchrony and metastability changes across the alpha and delta bands were standardized and averaged to form a single network dynamics composite. In the microstate domain, the extracted microstate metrics, including mean duration, Hurst exponent, global explained variance, GFP peak frequency, and graph density of significant transition probabilities, were standardized and averaged to form a single microstate composite. These three domain-level predictors were then entered sequentially into the hierarchical regression model. First, we regressed Δ*FM* on the spectral predictor. Next, we added the network-level dynamics composite to test whether it improved model fit beyond alpha power alone. Finally, we added the microstate composite to test whether microstate dynamics explained additional variance beyond the spectral and network-level predictors. Incremental model improvement was evaluated using nested model comparisons based on the change in explained variance and residual error between successive models. This approach was used to limit the number of predictors relative to the sample size and should be interpreted as an exploratory dimensionality-reduction strategy rather than as a data-driven selection of the strongest individual variable within each family.

**Table 1:** Hierarchical regression model comparison.

Model	*R* ^2^	Adjusted *R*^2^	*ΔR* ^2^	*F*-change	*p*-change
Alpha	0.526	0.494	–	–	–
Alpha + network dynamics	0.786	0.755	0.260	17.04	0.001
Alpha + network + microstate	0.789	0.740	0.003	0.16	0.700

Nested model comparisons showed that adding network-level dynamics significantly improved model fit beyond alpha power alone, whereas adding the microstate composite did not significantly improve the alpha + network model.

## 3 Results

### 3.1 Alpha peak shift and relative alpha power

Alpha peak frequency shifts have traditionally been proposed as markers of cortical reorganization during stroke recovery (Juhász et al., 2009). In our dataset, however, the maximum alpha peak shift across all channels did not exhibit a significant correlation with motor improvement (*r* = 0.09, *p* = 0.71). Change in relative alpha power averaged across channels showed a robust association with change in contralesional Fugl-Meyer score from Session 1 to Session 2 (*r* = 0.72, *p* = 0.00099). Figure 3 shows the spatial distribution of relative alpha power changes by presenting a topographical scalp map, where each electrode location is color-coded to reflect the mean difference in relative alpha power (Session 2 minus Session 1) across subjects. These findings suggest that while alpha frequency shifts alone may not reliably track functional gains, the proportion of power in the alpha band can provide a more sensitive index of neurophysiological changes linked to stroke rehabilitation. Because the main analyses focused on change scores, we also examined whether the primary spectral EEG measure was related to motor function at each individual session. Relative alpha power at the first session was significantly associated with baseline contralesional Fugl-Meyer motor score (*r* = 0.740, *p* = 0.00068). In contrast, the association between relative alpha power and contralesional Fugl-Meyer score at the three-month session was weaker and not statistically significant (*r* = 0.297, *p* = 0.246). These findings indicate that alpha power was related to early post-stroke motor status, and therefore the longitudinal alpha-recovery association should be interpreted in the context of baseline impairment.

**Figure 3:**
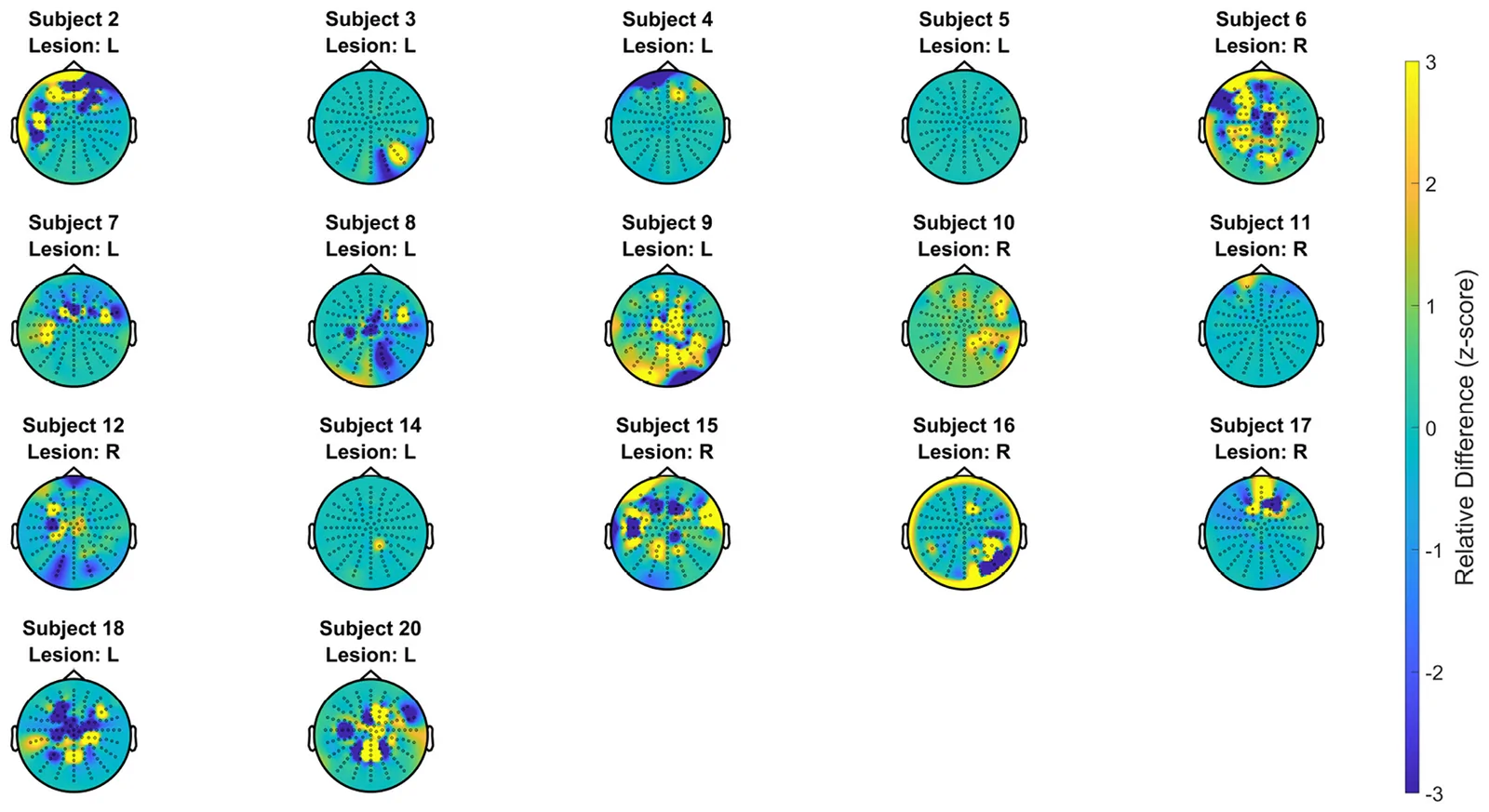
Spatial distribution of relative alpha power change. Topographic maps showing the change in relative alpha power from Session 2 to Session 1 for each individual subject. For each subject, relative alpha power was computed at each electrode as the ratio of alpha power to total power, and the difference between sessions (Session 2—Session 1) is visualized. Each map reflects the spatial pattern of alpha power modulation over time, with warmer colors indicating increases and cooler colors indicating decreases.

### 3.2 Metastability and synchrony

We examined the relationship between metastability, synchrony, and Δ*FM* scores across different frequency bands. In the delta band, metastability (*r* = 0.350) and synchrony (*r* = 0.315) showed moderate positive correlations with Δ*FM* score differences. Similar trends were observed in the theta band, where metastability (*r* = 0.356) and synchrony (*r* = 0.418) exhibited slightly stronger associations with Δ*FM*. The alpha band demonstrated the highest correlations, with metastability (*r* = 0.438) and synchrony (*r* = 0.417), suggesting that dynamic coordination in this frequency range may be more relevant to recovery. In contrast, the beta band showed weaker correlations, with metastability (*r* = 0.211) and synchrony (*r* = 0.246). While none of the correlations reached statistical significance at α = 0.05, the observed trends suggest increased metastability and synchrony in lower frequency bands. We note that, given its broad relative bandwidth (13–30 Hz, spanning more than an octave), the instantaneous phase derived for the beta band is less reliably interpretable than for the narrower delta, theta, and alpha bands, and low- and high-beta activity have been proposed to reflect functionally distinct rhythms (see Discussion, Limitations). For this reason, together with its comparatively weak and non-significant correlation with Δ*FM*, the beta-band estimate was not carried forward into the composite network index used in the Unified Multivariate Framework (Section 3.4).

### 3.3 Microstate analysis

All statistical measures extracted from the microstate time series were correlated with the Δ*FM*, and none of them individually reached significance at α = 0.05. Mean microstate duration (*r* = 0.09), Complexity (*r* = 0.05), GFP peaks (*r* = −0.18), and GEV (*r* = −0.15) all showed weak correlation relationships with Δ*FM*, suggesting that no single measure from microstates can be used to predict motor recovery solely. Since patients exhibited heterogeneous lesion locations, affected hemispheres, and recovery trajectories, the cortical reorganization was expected to vary greatly across individuals; hence, we performed subject-specific analyses of transition probabilities between different microstates. As shown in Supplementary Figure S1, there seem to be subject-specific changes in microstate transition probabilities between the sessions. Figure 2 illustrates a representative subject whose transition probabilities between Session 1 and Session 2 differed substantially. It should be noted that, in these figures, only transitions exceeding the baseline model are plotted; therefore, more arrows indicate more structured state-to-state transitions. To quantify these results, we computed the average graph density, which increased from 0.65 in Session 1 to 0.70 in Session 2. However, across subjects, the average graph density did not significantly correlate with Δ*FM* (*r* = 0.079, *p* = 0.76). The graph density is computed using the following equation:

$$\begin{array}{c} d=\frac{m}{n\left(n-1\right)} \end{array}$$ (6)

where *n* is the number of nodes and *m* is the number of edges in *G*. The number of connections in the graph is the same as the number of significant transition probabilities.

### 3.4 Unified multivariate framework

In the first-level regression, relative alpha power change alone accounted for 52.57% of the variance in motor recovery (*R*^2^ = 0.5257, adjusted *R*^2^ = 0.4941, overall model *p* = 0.0010). We then formally tested whether adding each subsequent predictor block improved model fit using nested model comparisons. Adding the network-level dynamics composite significantly improved the model beyond alpha power alone, increasing explained variance to 78.61% (adjusted *R*^2^ = 0.7555; Δ*R*^2^ = 0.2604, *F*(1, 14) = 17.04, *p* = 0.0010). By contrast, adding the microstate composite to the alpha + network model produced only a negligible and non-significant increase in explained variance (*R*^2^ = 0.7886, adjusted *R*^2^ = 0.7398; Δ*R*^2^ = 0.0025, *F*(1, 13) = 0.16, *p* = 0.700). These results indicate that network-level dynamics contributed significant explanatory information beyond alpha power in this sample, whereas the microstate composite did not significantly improve the model once alpha and network-level dynamics were included.

We next examined whether multicollinearity among the EEG predictors could account for the hierarchical regression results. Pairwise predictor correlations did not indicate severe collinearity: relative alpha power change was not significantly correlated with the network composite (*r* = −0.055, *p* = 0.835), the network and microstate composites showed a moderate but non-significant correlation (*r* = 0.385, *p* = 0.127), and the alpha and microstate composites showed a moderate non-significant correlation (*r* = −0.444, *p* = 0.074). Variance inflation factors were low for all predictors (alpha = 1.27, network = 1.20, microstate = 1.49), indicating that multicollinearity was not a major concern in the model. Simple predictor-outcome correlations showed that relative alpha power change was significantly associated with Δ*FM* (*r* = 0.725, *p* = 0.001), the network composite showed a moderate association that approached significance (*r* = 0.470, *p* = 0.057), and the microstate composite was not associated with Δ*FM* (*r* = −0.096, *p* = 0.713).

## 4 Discussion

In this study, we examined how EEG metrics of global dynamical properties (as measured by metastability and synchrony), spatiotemporal features (as measured by microstate analysis), and classical spectral measures (particularly alpha band power) relate to post-stroke changes in Fugl-Meyer scores (Δ*FM*). Of these, only the relative alpha band power showed a consistent positive association with (Δ*FM*). Metastability and synchrony demonstrated moderate positive correlations, especially in the alpha and theta bands, suggesting that enhanced large-scale oscillatory coordination may favor better recovery outcomes. In this study, only spectral and network-level features demonstrated clear associations with recovery outcomes. Microstate measures, including duration, global explained variance (GEV), complexity, GFP peak frequency, and transition probability measures, showed low or inconsistent correlations and did not contribute meaningfully to explained variance. Thus, our data do not support strong conclusions regarding the role of microstate dynamics in stroke recovery. Overall, these findings suggest that stroke recovery primarily involves reestablishing functional balance at the spectral and network levels of brain organization. In our dataset, improvements in recovery outcomes were associated with longitudinal increases in spectral power in the alpha range and elevated measures of network metastability and synchrony. These findings suggest that elevations in spectral and network-level dynamics may reflect ongoing reorganization processes during motor recovery. We did not observe statistically meaningful contributions of microstate dynamics in this cohort. Overall, these exploratory observations indicate that stroke recovery may be accompanied by enhanced spectral and network properties, though stronger evidence would be required to establish causal relationships or normative thresholds.

Our finding of a positive correlation between relative alpha power increases and motor recovery appears to conflict with reports that suggest higher alpha at single time points portends worse function (Leonardi et al., 2022; Martino Cinnera et al., 2025; Sebastián-Romagosa et al., 2020; Hoshino et al., 2020). Importantly, our metric captured longitudinal change in alpha rather than its absolute level at a single time point. The literature on acute stroke severity typically emphasizes global slowing, increased delta and theta power alongside reduced alpha and beta power—rather than excess alpha, with indices such as the delta/alpha ratio well established as markers of stroke severity (Sheorajpanday et al., 2011). We therefore interpret our finding not as a reversal of a static “excess alpha” pathology, but as reflecting a longitudinal normalization of spectral power away from the slowed, low-alpha profile characteristic of the acute phase, consistent with reports that a shift from slow-wave toward alpha/beta-dominant activity during rehabilitation is associated with greater motor recovery (Trujillo et al., 2017). In fact, recent longitudinal studies support this interpretation: patients who show a shift from slow-wave activity toward alpha-beta activity during rehabilitation tend to achieve greater recovery (Trujillo et al., 2017), in other words, the reduction of EEG slowing (quantified by a flattening of the spectral exponent) was significantly correlated with improved motor function (Lanzone et al., 2022). Thus, increases in alpha power over the subacute period may signify the restoration of more normal network excitability and engagement, rather than pathological inhibition (Sebastián-Romagosa et al., 2020). Future research with larger cohorts should clarify the precise role of alpha-band dynamics in post-stroke motor recovery and whether any early EEG spectral indicators—e.g. alpha laterality indices or delta/alpha ratios—have true prognostic value when measured in the acute phase of the stroke.

It has been reported that stroke patients with lower motor function showed prolonged microstates of types A and B compared to controls, while those microstate parameters tended to normalize as function improved (Hao et al., 2022). Similarly, it has been reported that certain microstate metrics (e.g. fractional coverage of specific classes) were significantly correlated. In our analyses, microstate features showed limited unique contribution once spectral power and large-scale network dynamics were considered. This suggests that much of the variance captured by microstate metrics overlaps with broader dynamical measures, whereas alpha power and network-level synchrony/metastability emerged as the main independent contributors to model performance.

The absence of a robust microstate—recovery relationship in our data also invites a reconsideration of what microstate parameters are likely to index in a stroke population. Microstate analysis was developed largely in healthy cohorts and in conditions characterized by distributed rather than focal pathology, such as psychiatric and neurocognitive disorders (Tait et al., 2020; Musaeus et al., 2019), and its extension to focal structural lesions raises both methodological and interpretive questions. Methodologically, a focal lesion alters local tissue conductivity, which can, in principle, distort scalp topography for sources near the lesion boundary and thereby bias the microstate maps derived from that topography (Piai et al., 2024). We attempted to mitigate this by fitting microstate templates individually for each subject and session rather than imposing a common group template, allowing lesion-specific topographical idiosyncrasies to be absorbed into each patient's own maps; however, this does not eliminate the underlying conductivity concern, and we flag it as a limitation of applying microstate analysis to structurally heterogeneous, lesioned brains. Interpretively, it is also plausible that microstate parameters are better suited to indexing lesion burden or injury severity than tracking longitudinal recovery. Prior prognostic value has been reported chiefly for acute-phase microstate patterns rather than for their change over time (Zappasodi et al., 2017), which is more consistent with microstates functioning as a marker of injury state than as a marker of the recovery process itself. Relatedly, microstate classes have established associations with non-motor networks: class C is linked to self-referential and salience processing, and class D to attentional and cognitive control networks (Britz et al., 2010; Michel and Koenig, 2018), rather than to motor circuits specifically. Given that our outcome measure was a motor scale, the limited microstate associations observed here may partly reflect a mismatch between what microstates preferentially index and what Δ*FM* captures, rather than a true absence of stroke-related microstate reorganization. Notably, microstates dynamics are comparatively well established as biomarkers in mood and neurocognitive disorders (Chivu et al., 2024), which are highly prevalent after stroke, affecting an estimated one quarter to one third of patients (Ayerbe et al., 2013). As mood and cognitive outcomes were not assessed in this dataset, we were unable to test this association directly, but it represents a plausible alternative target for microstate biomarkers in stroke populations and a natural direction for future work.

Neurophysiologically, increased metastability and synchrony were correlated with recovery outcomes in our dataset, potentially reflecting a greater degree of freedom for state transitions, indicating that the system state space is more accessible. While the functional significance of these associations cannot be directly discussed from these analyses, it is possible that elevated metastability supports the exploration of a broader repertoire of network configurations, which may in turn facilitate neuroplastic adaptation and functional reorganization in motor-related circuits following stroke. In parallel, we note that microstate duration and GEV numerically decreased alongside recovery in our data, which would be consistent with greater flexibility in neural network organization; however, as these correlations were not statistically significant (Section 3.3), this observation is offered only as a descriptive pattern for future hypothesis generation, not as evidence of a functional relationship between microstate dynamics and recovery-related network reorganization. Although metastability has been conceptually linked to measures such as the Kuramoto order parameter, no existing studies have directly examined the relationship between the Kuramoto parameter and Fugl-Meyer outcomes. Future work can explore the Kuramoto order parameter to better understand how oscillatory coupling supports motor recovery. Our results are broadly consistent with previous findings identifying large-scale neural coordination as relevant to stroke rehabilitation, although Kawano et al. (2017) and Riahi et al. (2020) quantified synchrony as a pairwise measure between electrodes rather than the global order-parameter index used here. In the Unified Multivariate Framework, relative alpha power changes emerged as the single strongest predictor of motor recovery, explaining 52.57% of the variance; however, adding global network-level features and spatiotemporal microstate metrics incrementally raised the explained variance first to 78.61% and then to 78.86%. This pattern indicates that these different EEG domain features capture distinct aspects of post-stroke cortical reorganization. The corrected nested model comparisons showed that network-level dynamics significantly improved model fit beyond alpha power alone, whereas the microstate composite did not add significant explanatory value after alpha power and network dynamics were included. Therefore, the multivariate findings should be interpreted as evidence for an incremental contribution of network-level dynamics in this exploratory cohort, but not for an independent contribution of microstate metrics. It should be noted that since the sample size was limited in this study, a composite index for each domain was created by averaging standardized microstate metrics or network-level measures to overcome overfitting issues. Although this domain-level aggregation potentially sacrifices some granularity, it preserves statistical stability in a small-sample setting. Future studies with larger cohorts could more fully exploit the diversity of microstate and network features, offering deeper insight into the mechanisms of stroke recovery.

Some prior studies have reported EEG features with modest prognostic utility. Connectivity measures at four weeks were shown to predict motor scores at 8 weeks (Hoshino et al., 2020; Riahi et al., 2020; Lee et al., 2022), and certain microstate parameters measured acutely were found to relate to recovery at 5 months (Zappasodi et al., 2017). Based on these observations and the current findings, it is suggested that future large-scale or longitudinal studies systematically assess the prognostic value of integrated EEG biomarkers using both large-scale EEG network dynamics and spatiotemporal patterns combined with traditional spectral features, particularly in the acute—subacute transition. Solely based on the current results, it remains unclear whether these measures simply reflect epiphenomenal changes or play a causal role in enabling motor recovery.

Despite these promising findings, several limitations should be acknowledged. A primary limitation is the small sample size, which may significantly limit the statistical power and the generalisability of our results. Future studies with larger cohorts are necessary to confirm the robustness and reproducibility of these correlations. It is also possible that the absence of strong associations with microstate features reflects underlying heterogeneity in lesion location or stroke subtype; future work incorporating lesion-informed subgrouping or mixed-effects models may help clarify these relationships. This heterogeneity in lesion side, vascular territory, and stroke severity (NIHSS range 3–27) further motivates the caution urged above regarding the interpretation of microstate findings in a structurally heterogeneous sample, and may also contribute to variability in the spectral and network-level measures. Relatedly, our outcome measure was restricted to motor recovery as indexed by the Fugl-Meyer scale; given that microstates are more consistently linked to attentional, salience, and self-referential networks than to motor networks, a cognitive, mood, or broader functional outcome measure may be more sensitive to microstate-related variance, particularly in a small-sample design where a strict motor bias in the outcome could obscure genuine but non-motor signal. A further limitation is that the longitudinal design included only two measurement time points. We therefore analyzed associations between EEG and motor recovery using change scores, which directly capture the session-to-session change of interest but cannot model individual recovery trajectories in detail. Future studies with more frequent longitudinal assessments would allow repeated-measures correlation or mixed-effects modeling approaches to better characterize within-participant EEG-behavior relationships over time. The present findings should also be interpreted in light of baseline EEG-behavior relationships. Relative alpha power was significantly associated with motor status at the first session, indicating that the observed relationship between alpha change and motor recovery may partly reflect baseline impairment or recovery potential. Because the study included only two time points, we could not fully separate baseline status, spontaneous recovery trajectory, and within-participant EEG-behavior coupling. Additionally, our metastability and synchrony analyses relied on a sequential (order-dependent) orthogonalization step intended to reduce electrode-level spatial leakage prior to phase extraction; this procedure is not symmetric with respect to channel order, and its necessity and validity for phase-based measures specifically—as opposed to the amplitude-based connectivity measures for which such leakage-correction methods were originally developed and validated—has not been established in the literature (Castaldo et al., 2023). Future work should assess the sensitivity of KOP-based metastability and synchrony estimates to this and alternative (e.g., symmetric or no) leakage-correction procedures.

We also note that our beta band (13–30 Hz) spans a wide frequency range relative to its center frequency, and the instantaneous phase derived via the Hilbert transform is only well defined for approximately narrowband signals; pooling frequencies that may reflect functionally distinct rhythms—as has been proposed for low- and high-beta specifically—into a single phase estimate may obscure band-specific effects or yield an ill-defined composite phase. We report this beta-band result descriptively for completeness, but for this reason, together with its weak and non-significant correlation with recovery, it was excluded from the composite network index used in our multivariate model, which relied on the narrower and better-defined delta and alpha bands.

## 5 Conclusion

In conclusion, our findings suggest that in this cohort, longitudinal increases in alpha power, along with enhanced metastability and synchrony, were associated with better post-stroke motor recovery. Microstate features provided minimal independent predictive value in this cohort. Together with prior research, these results are consistent with a link between adaptive neural coordination, spectral dynamics, and motor recovery after stroke, although the correlational and observational nature of this study cannot establish whether these EEG signatures contribute causally to recovery, whether they instead reflect the extent of the underlying injury, or whether they are themselves a downstream consequence of the recovery process. The unified model accounted for a substantial proportion of variability in post-stroke motor recovery, underscoring its potential as an integrative biomarker framework for stroke rehabilitation. Future research should explore whether these EEG-derived measures, individually or in combination, can serve as reliable biomarkers to predict stroke rehabilitation outcomes and guide targeted therapeutic interventions.

## Data Availability

The data analyzed in this study is subject to the following licenses/restrictions: The dataset analysed in this study is not publicly available due to data protection restrictions under the GDPR. The data were provided through the AISN consortium under a controlled data-sharing agreement with the principal investigator. Requests for access may be considered on reasonable request and subject to consortium approval, institutional agreements, and applicable ethical and legal regulations. Requests to access these datasets should be directed to adrian.guggisberg@hug.ch.
