# Supplementary material for: Electrophysiological signature of stroke recovery: investigating EEG biomarkers for prognostic insights
Source: Front Stroke. 2026 Sep 28;5:1894659. doi: 10.3389/fstro.2026.1894659 (PMC13654781; doi:10.3389/fstro.2026.1894659)
Supplement: Supplementary file 1 [file Supplementary_File_1.pdf]

Supplementary Material

Electrophysiological signature of stroke recovery: investigating EEG biomarkers for prognostic insights

Khalili-Ardali M, Sharma V, Mandahar TS, Páscoa dos Santos F, Tiesinga P and Ramsey N

1 Supplementary Material

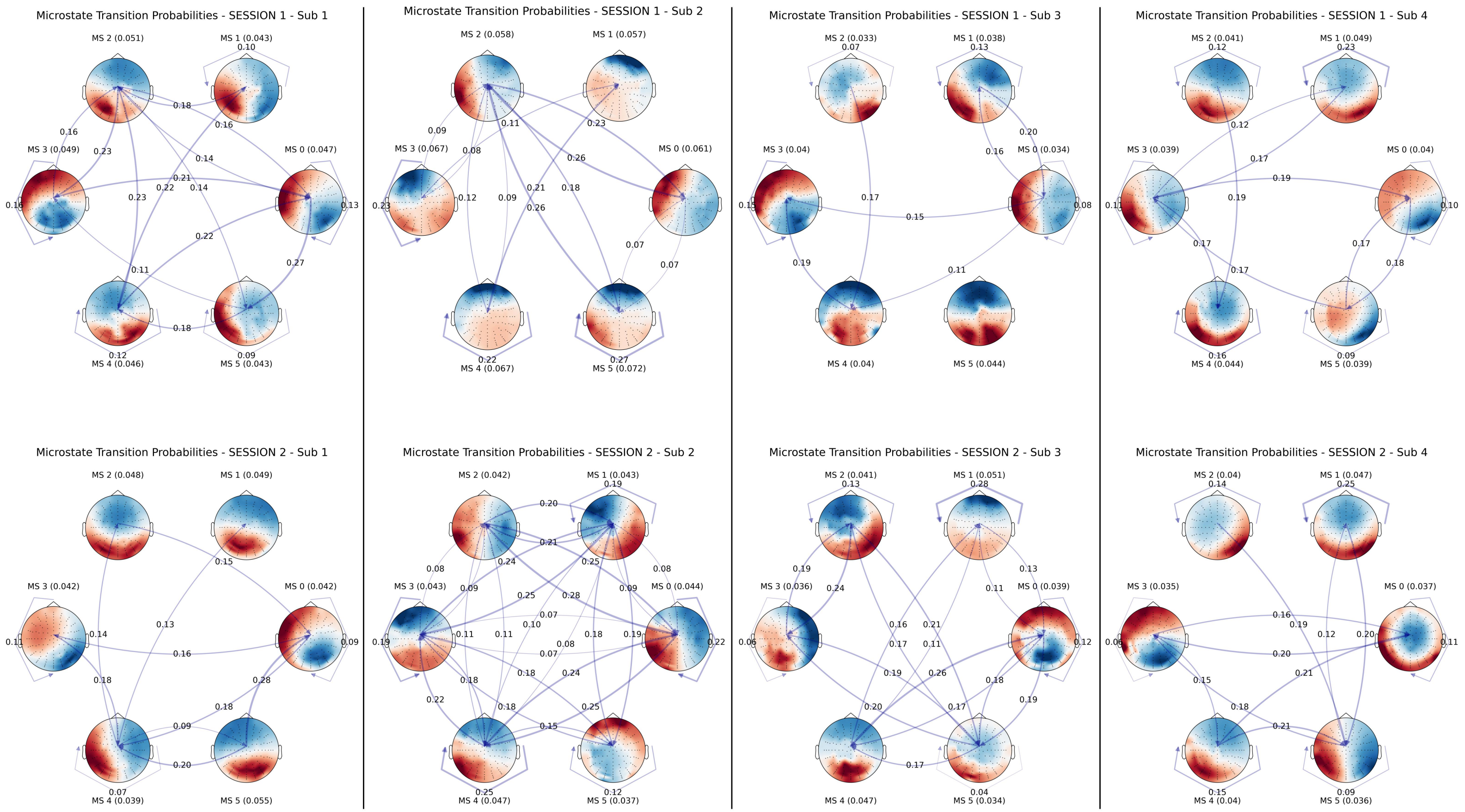
Microstate Transition Probabilities - SESSION 1 - Sub 1
MS 2 (0.051)
MS 1 (0.043)
MS 3 (0.049)
MS 0 (0.047)
MS 4 (0.046)
MS 5 (0.043)
Microstate Transition Probabilities - SESSION 1 - Sub 2
MS 2 (0.058)
MS 1 (0.057)
MS 3 (0.067)
MS 0 (0.061)
MS 4 (0.067)
MS 5 (0.072)
Microstate Transition Probabilities - SESSION 1 - Sub 3
MS 2 (0.033)
MS 1 (0.038)
MS 3 (0.04)
MS 0 (0.034)
MS 4 (0.04)
MS 5 (0.044)
Microstate Transition Probabilities - SESSION 1 - Sub 4
MS 2 (0.041)
MS 1 (0.049)
MS 3 (0.039)
MS 0 (0.04)
MS 4 (0.044)
MS 5 (0.039)
Microstate Transition Probabilities - SESSION 2 - Sub 1
MS 2 (0.048)
MS 1 (0.049)
MS 3 (0.042)
MS 0 (0.042)
MS 4 (0.039)
MS 5 (0.055)
Microstate Transition Probabilities - SESSION 2 - Sub 2
MS 2 (0.042)
MS 1 (0.043)
MS 3 (0.043)
MS 0 (0.044)
MS 4 (0.047)
MS 5 (0.037)
Microstate Transition Probabilities - SESSION 2 - Sub 3
MS 2 (0.041)
MS 1 (0.051)
MS 3 (0.036)
MS 0 (0.039)
MS 4 (0.047)
MS 5 (0.034)
Microstate Transition Probabilities - SESSION 2 - Sub 4
MS 2 (0.04)
MS 1 (0.047)
MS 3 (0.035)
MS 0 (0.037)
MS 4 (0.04)
MS 5 (0.036)

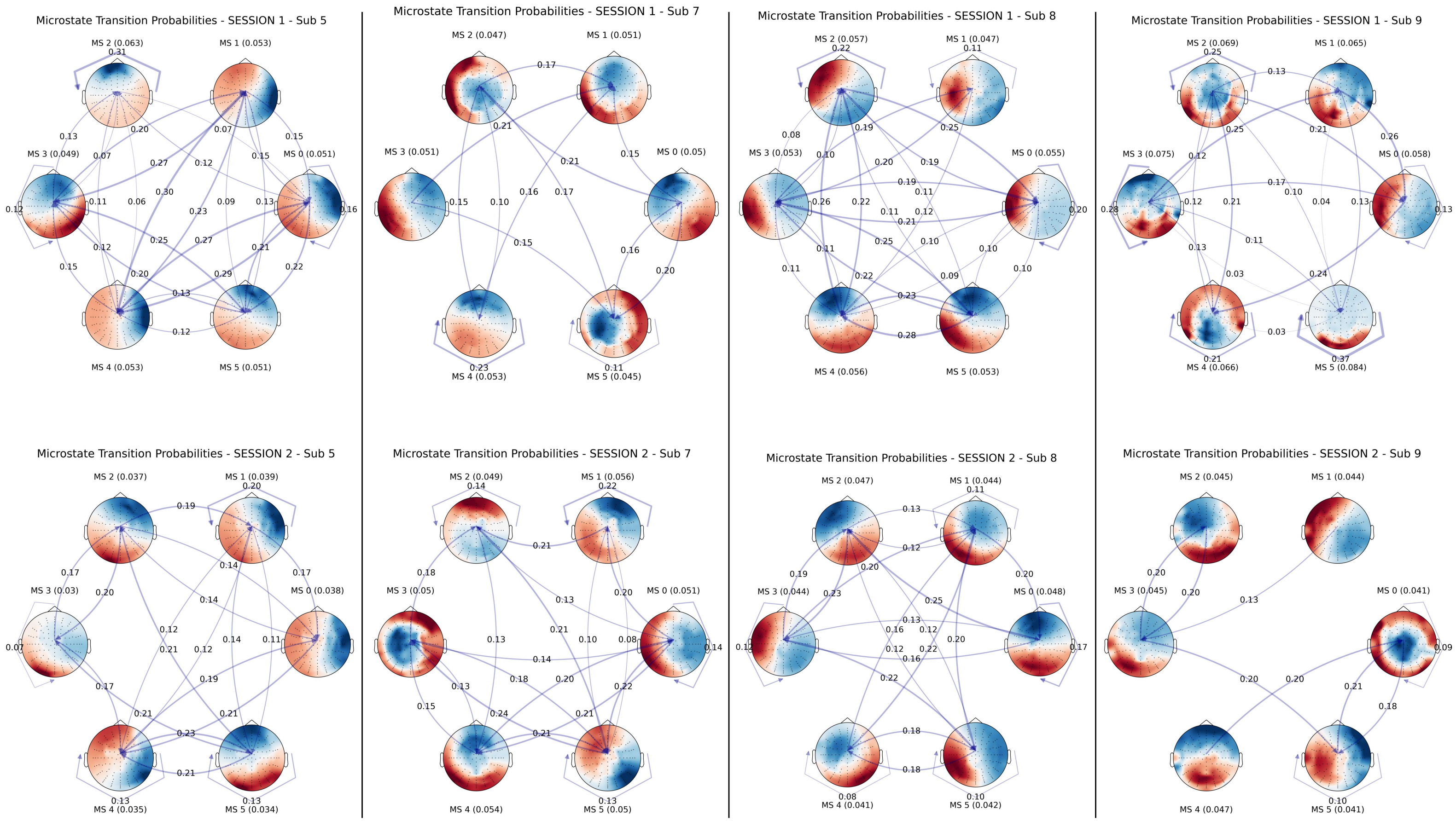

Microstate Transition Probabilities - SESSION 1 - Sub 5
Microstate Transition Probabilities - SESSION 1 - Sub 7
Microstate Transition Probabilities - SESSION 1 - Sub 8
Microstate Transition Probabilities - SESSION 1 - Sub 9
Microstate Transition Probabilities - SESSION 2 - Sub 5
Microstate Transition Probabilities - SESSION 2 - Sub 7
Microstate Transition Probabilities - SESSION 2 - Sub 8
Microstate Transition Probabilities - SESSION 2 - Sub 9

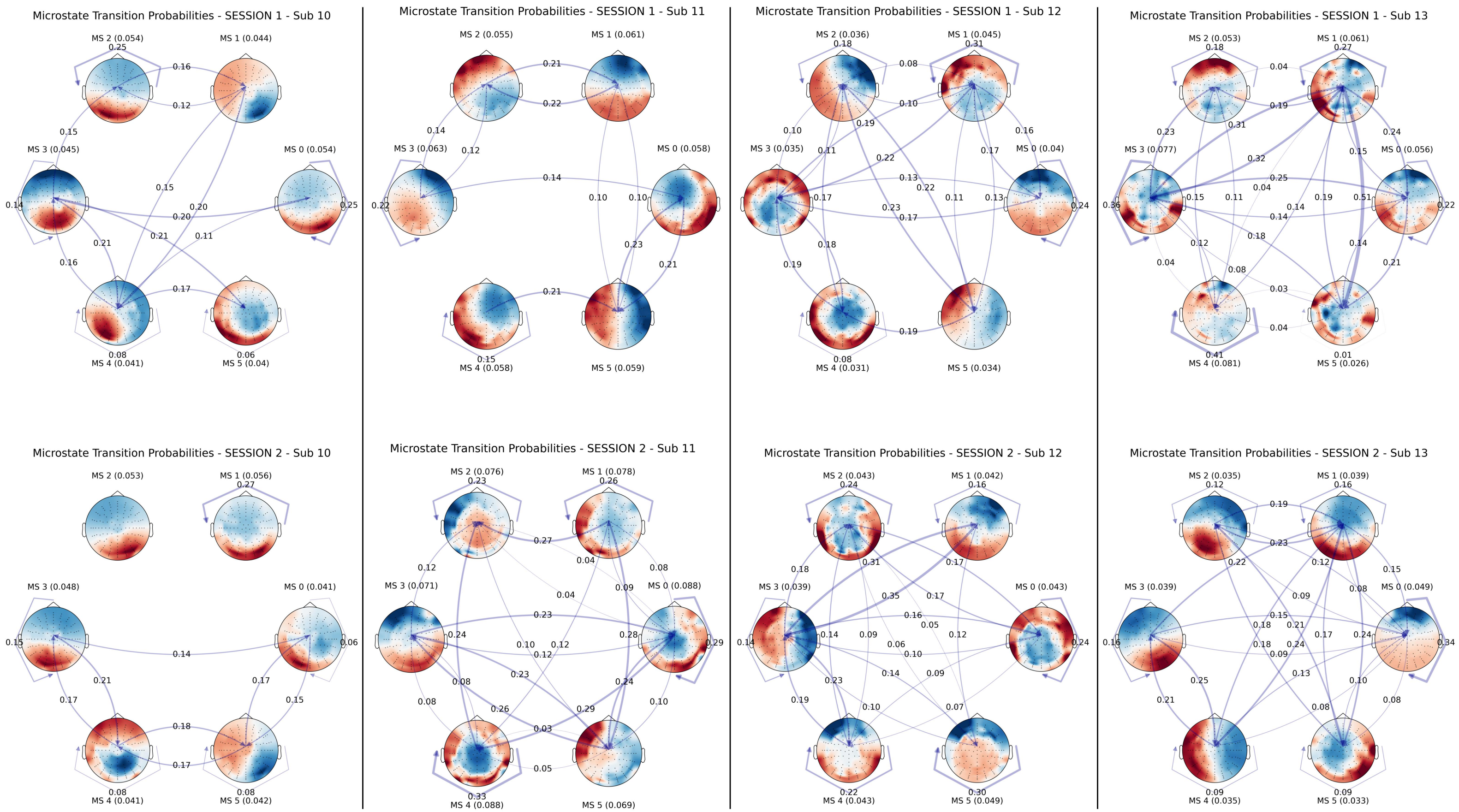

Microstate Transition Probabilities - SESSION 1 - Sub 10
MS 2 (0.054)
MS 1 (0.044)
MS 3 (0.045)
MS 0 (0.054)
MS 4 (0.041)
MS 5 (0.04)
Microstate Transition Probabilities - SESSION 1 - Sub 11
MS 2 (0.055)
MS 1 (0.061)
MS 3 (0.063)
MS 0 (0.058)
MS 4 (0.058)
MS 5 (0.059)
Microstate Transition Probabilities - SESSION 1 - Sub 12
MS 2 (0.036)
MS 1 (0.045)
MS 3 (0.035)
MS 0 (0.04)
MS 4 (0.031)
MS 5 (0.034)
Microstate Transition Probabilities - SESSION 1 - Sub 13
MS 2 (0.053)
MS 1 (0.061)
MS 3 (0.077)
MS 0 (0.056)
MS 4 (0.081)
MS 5 (0.026)
Microstate Transition Probabilities - SESSION 2 - Sub 10
MS 2 (0.053)
MS 1 (0.056)
MS 3 (0.048)
MS 0 (0.041)
MS 4 (0.041)
MS 5 (0.042)
Microstate Transition Probabilities - SESSION 2 - Sub 11
MS 2 (0.076)
MS 1 (0.078)
MS 3 (0.071)
MS 0 (0.088)
MS 4 (0.088)
MS 5 (0.069)
Microstate Transition Probabilities - SESSION 2 - Sub 12
MS 2 (0.043)
MS 1 (0.042)
MS 3 (0.039)
MS 0 (0.043)
MS 4 (0.043)
MS 5 (0.049)
Microstate Transition Probabilities - SESSION 2 - Sub 13
MS 2 (0.035)
MS 1 (0.039)
MS 3 (0.039)
MS 0 (0.049)
MS 4 (0.035)
MS 5 (0.033)

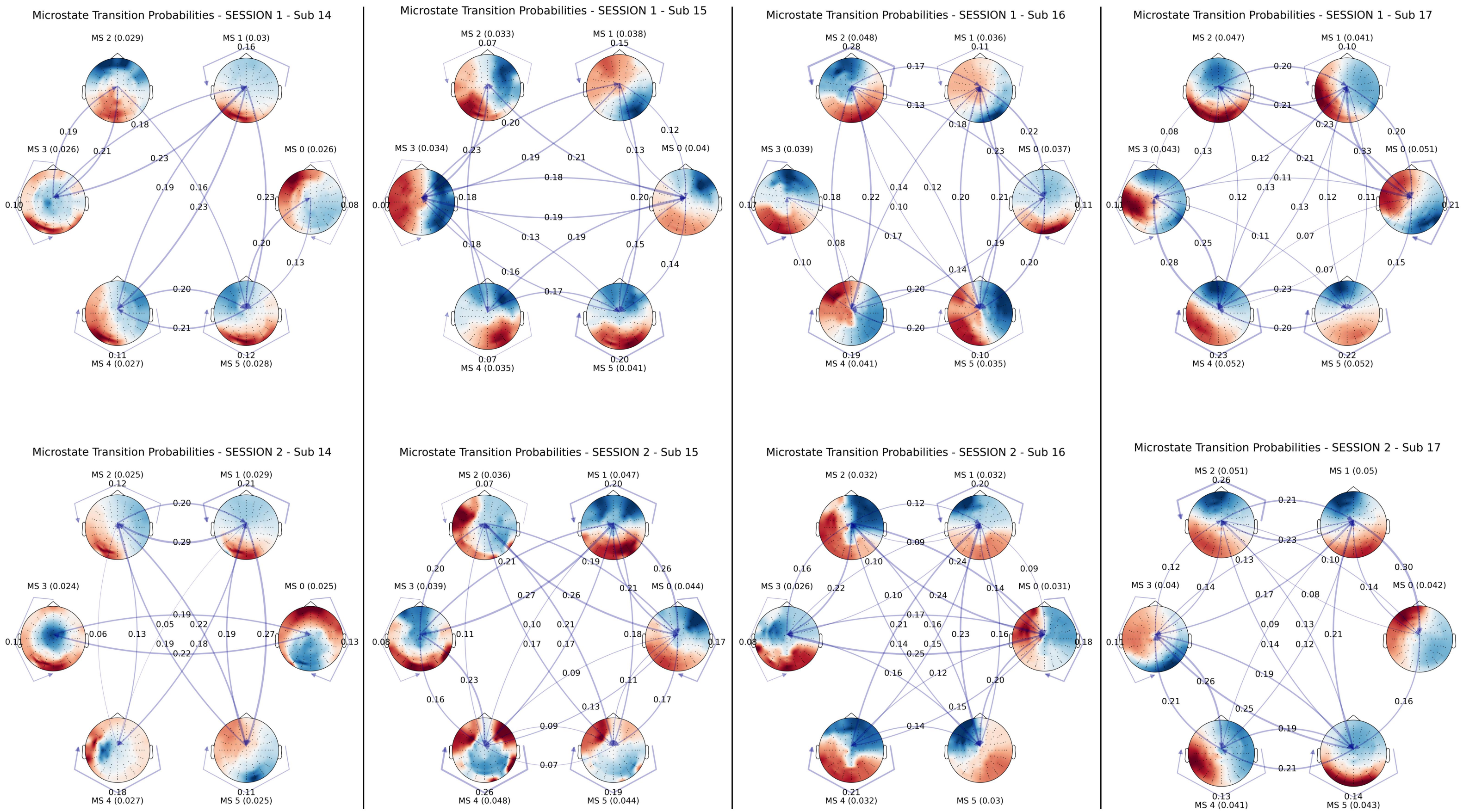

Figure S1: Transition Probabilities for all subjects (columns) and sessions (rows)
